# **Water-insoluble exopolysaccharide synthesized by glucosyltransferases mediates the antibacterial activity of ClyR against** ***Streptococcus mutans***

**DOI:** 10.1080/20002297.2025.2566894

**Published:** 2025-10-09

**Authors:** Qizhao Ma, Xiaowan Wang, Mai Xu, Ziyi Yang, Dian Zhang, Jiamin Chen, Tao Gong, Hang Yang, Yuqing Li

**Affiliations:** a State Key Laboratory of Oral Diseases, National Center for Stomatology, National Clinical Research Center for Oral Diseases, West China Hospital of Stomatology, Sichuan University, Chengdu, China; b Department of Pediatric Dentistry, West China Hospital of Stomatology, Sichuan University, Chengdu, China; c State Key Laboratory of Virology and Biosafety, Wuhan Institute of Virology, Chinese Academy of Sciences, Wuhan, China; d University of Chinese Academy of Sciences, Beijing, China; e Hubei Jiangxia Laboratory, Wuhan, China

**Keywords:** Dental caries, *Streptococcus mutans*, biofilm, ClyR, water-insoluble EPS

## Abstract

**Background:**

Dental caries is a widespread global health issue strongly associated with *Streptococcus mutans*. Bacteriophage-derived lytic enzymes such as ClyR hold considerable promise as antibacterial potential, but the molecular mechanisms underlying their activity against *S. mutans* remain unclear.

**Objective:**

This study aimed to determine the role of water-insoluble exopolysaccharides (EPS) in mediating the antibacterial activity of ClyR against *S. mutans*.

**Design:**

We compared the antibacterial effects of ClyR on *S. mutans* UA159 and its Δ*gtfB* mutant, which is characterized by reduced synthesis of water-insoluble EPS. Biofilm architecture and susceptibility were assessed using scanning electron microscopy, confocal laser scanning microscopy, and biomass quantification. Adsorption assays were conducted to evaluate the interaction between ClyR and water-insoluble EPS.

**Results:**

The Δ*gtfB* mutant exhibited significantly higher resistance to ClyR than* S. mutans* UA159, with reduced biofilm disruption and bacterial loss after treatment. *In vitro* assays confirmed that water-insoluble EPS specifically adsorbed ClyR, with binding localized to its catalytic PlyCAC domain.

**Conclusions:**

Water-insoluble EPS synthesized by *S. mutans* glucosyltransferases plays a critical role in modulating bacterial susceptibility to ClyR. These findings reveal a novel mechanism underlying bacteriophage lysin activity and highlight EPS as a potential target for enhancing ClyR efficacy against cariogenic biofilms.

## Introduction

Dental caries, characterized by dental enamel demineralization, remains a major global oral health challenge. *Streptococcus mutans* is central to the etiology of dental caries due to its virulence factors, including acid tolerance, adhesive ability, and the synthesis of extracellular polysaccharides [1–3]. Advanced molecular techniques, such as fluorescence *in situ* hybridization (FISH) applied to plaque samples from carious sites, have unequivocally confirmed the central role of *S. mutans* in cariogenic biofilm formation [4], underlining its pivotal role in the caries process.

The therapeutic potential of bacteriophage lytic enzymes, or endolysins, has attracted attention due to their specificity and efficacy in lysing bacterial cell walls [5]. These enzymes, characterized by a catalytic domain (CD) and a cell binding domain (CBD), are integral to bacteriophage-mediated bacterial lysis at the end of the phage replication cycle [6,7]. Early research by Freimer [8] on the application of lytic enzymes to treat streptococcal infections set the stage for their broader therapeutic use. Protein engineering has significantly expanded this potential, allowing the synthesis of chimeric lysozymes that combine lytic domains from diverse sources [9]. These engineered constructs possess enhanced lytic breadth, activity, and stability [10,11], marking a promising frontier in the battle against infectious diseases.

ClyR is a bioengineered lysin composed of the CHAP (Cysteine, Histidine-Dependent Amidohy-Drolase/Peptidase) catalytic domain from PlyC (PlyCAC), a lysin produced by a *Streptococcus pyogenes* phage with a broad host range against *Streptococcus* species, and the cell wall binding domain from PlySs2 (PlySb), which is derived from a *Streptococcus suis* phage and also exhibits broad-spectrum lytic activity. By integrating these domains, ClyR was designed to enhance lytic potency and spectrum against pathogenic streptococci [12–15]. This discovery heralded ClyR as an innovative bacteriophage chimeric lysozyme with activity against *S. mutans*, the primary bacterial species implicated in dental caries. However, variability in the susceptibility of *S. mutans* clinical isolates to ClyR poses a challenge to its clinical application, underscoring the need to investigate the factors that influence bacterial resistance or sensitivity to this promising antimicrobial agent [16]. The present study is a research endeavor to unravel the mechanisms underlying the varied susceptibility of *S. mutans* to ClyR, deepen the understanding of ClyR's antibacterial action, and provide insights that may inform the development of future strategies for the prevention and management of dental caries, thereby contributing valuable knowledge to the field of microbial pathogenesis and therapeutic interventions.

## Materials and methods

### Bacterial strains and growth conditions

The *S. mutans* UA159 strain was obtained from the American Type Culture Collection (ATCC) (Manassas, VA, USA), and its derivative was provided by the State Key Laboratory of Oral Diseases at Sichuan University. *S. mutans* UA159 and its derivative were routinely cultured in brain heart infusion (BHI) broth (BD, USA). Monoclonal clones (single-colony isolates) were obtained by streaking *S. mutans* strains onto brain heart infusion agar (BHIA) plates and selecting individual well-isolated colonies. All strains were incubated under anaerobic conditions (80% N₂, 10% CO₂, 10% H₂) to simulate the oxygen-limited environment characteristic of mature dental biofilms. For biofilm assays, BHI supplemented with 1% sucrose (Sigma; w/v) was used as BHIS for culturing biofilms. Bacterial growth was monitored by measuring the optical density at 600 nm (OD_600_). For all bactericidal assays, including both planktonic and biofilm experiments, ClyR was used at a final concentration of 300 µg/mL. Planktonic cultures were incubated with ClyR for 1 or 7 h to capture the kinetics of bacterial killing, while biofilm samples were treated with ClyR for 24 h to ensure effective exposure within the biofilm matrix and allow for sufficient antimicrobial activity against sessile cells.

### Expression and purification of ClyR, PlyCAC and ClyF proteins

ClyR, PlyCAC, and ClyF protein expression strains were constructed previously [12,17]. *Escherichia coli* cells were cultured to an OD_600_ of 0.6−0.8, induced by 0.2-mM isopropyl-β-D-thiogalactoside, and grown for 12 h at 16 °C. The bacterial cells were lysed by a high-pressure cell cracker in PBS at pH = 7.4. The supernatant was filtered through a 0.45-μm filter and passed through a GSTSep glutathione agarose resin column pre-equilibrated with PBS. After washing with cleavage buffer [50-mM Tris–HCl (pH = 7.0), 0.15-M NaCl, 1-mM EDTA, 1-mM DTT], 2 U PreScission protease was added per 100-μg GST-tag protein and incubated at 4 °C for 12 h and eluted with cleavage buffer. Collected proteins were dialyzed against PBS and finally stored at 4 °C after filtration. Protein concentration was determined by bicinchoninic acid assay (Pierce Rockford, IL, USA), using BSA as the standard.

### Construction of *S. mutans* Δ*gtfB* mutant

The in-frame deletion mutant Δ*gtfB* of *S. mutans* UA159 was generated using a two-step transformation protocol as described previously [18]. The homologous sequences of approximately 1 kb upstream and downstream of the *gtfB* open reading frame were amplified using specific primers. Subsequently, the IFDC2 cassette, a laboratory-generated non-polar erythromycin resistance and *p*-Cl-Phe sensitivity cassette, was PCR-amplified using IFDC2F/IFDC2R primers [18]. The resulting three PCR amplicons were assembled by overlap extension PCR, yielding a 4.5-kb fragment that was subsequently transformed into *S. mutans* UA159 cells. Transformants were selected on BHI plates supplemented with erythromycin at a concentration of 12 μg/mL to facilitate the identification of positive clones. In the second transformation step, PCR amplification was used to generate upstream and downstream fragments corresponding to the *gtfB* open reading frame, which were subsequently overlapped to create an upstream-downstream amplicon. This DNA construct was then introduced into the strain obtained from the first step via transformation using BHI selection plates containing *p*-Cl-Phe (Sigma) at a 4-mg/mL concentration. The constructed deletion mutant was further confirmed through PCR analysis and sequencing. All primers used in this study are listed in Supplementary Table S1.

### Bactericidal assays

A series of experiments were conducted to investigate the lytic conditions of ClyR against *S. mutans*. Overnight cultures of *S. mutans* UA159 were diluted 1:10 into fresh BHI medium and further diluted 10-, 50-, and 100-fold when OD_600_ reached 0.5. In a 96-well plate, 100 µL of different concentrations (range: 50–500 µg/mL) of ClyR solution in PBS and an equal volume of multiple dilutions of bacterial solution were added, followed by incubation at 37 °C for different durations (10 min, 20 min, 30 min, etc.). At the end of the incubation period, 10 µL of the mixture was dropped onto BHIA plates after a 10-fold gradient dilution. Based on colony growth, the plates were then incubated at 37 °C for 48 h for colony-forming unit (CFU) counting.

### SEM and CLSM analyses of biofilms

*S. mutans* biofilms were observed under a scanning electron microscope (SEM) (FEI, Hillsboro, OR, USA). Bacteria diluted after overnight growth were further diluted 1:100 into BHIS at OD = 0.5 and then added to a 24-well plate with a sterile glass sheet placed at the bottom for 24 h of anaerobic incubation. The experimental group was treated with ClyR, while the control group was treated with an equivalent dose of PBS. The treated biofilms were rinsed three times with sterile PBS and fixed overnight with 2.5% (v/v) glutaraldehyde, followed by storage at 4ºC for 16 h. Subsequently, they were washed again with PBS and dehydrated using a gradient concentration of alcohol. The samples were observed by SEM (×20,000 magnification).

The biofilm architecture was observed using a confocal laser scanning microscope (CLSM) (Nikon, *N*-SIM). To label the extracellular polymeric substances, 1-µM Alexa Fluor 647 dextran conjugate (Life Technologies, Grand Island, NY, USA) was added to BHIS before inoculation. The bacterial solution in the mid-logarithmic growth phase was diluted 1:100 into BHIS and then added to the bottom of a glass dish for incubation for 24 h. After incubation, the biofilms were rinsed with 0.9% NaCl three times and stained with 2.5-µM SYTO 9 (Life Technologies) for 15 min to label bacterial cells. Biofilm images were captured and collected using a ×40 objective lens at a wavelength range of 655−690 nm for Alexa Fluor 647 and 495−515 nm for SYTO 9 fluorescence signals. Three randomly selected areas within each biofilm were scanned. ImageJ software (National Institutes of Health, USA) was used to calculate biofilm thickness and biomass.

### Zymogram assays

The cells of *S. mutans* UA159 and its derivatives were pelleted in the mid-logarithmic phase by centrifugation (4000 g, 4 °C, 10 min). The resulting supernatant was mixed with approximately one-third of its volume of absolute ethanol and immediately frozen at −80 °C for 30 min. After another round of centrifugation (25000 rpm, 4 °C, 15 min), the pellet was resuspended in 100 μL of PBS to obtain Gtfs. To visualize the expression level of Gtfs, proteins were separated using a 6% SDS-PAGE gel after quantification with a BCA protein quantification kit (Beyotime, China) to ensure equal total protein amounts across samples. The Gtfs proteins were renatured using the 0.2-M sodium phosphate buffer containing 2.5% Triton X-100 to assess their activity. Subsequently, the gel was transferred into 0.2-M sodium phosphate buffer containing 0.2% (w/v) T-70 glucan and 5% (w/v), followed by overnight incubation at 37 °C to observe water-insoluble EPS content on the gel.

### Synthesis of EPS *in vitro*

We synthesized water-insoluble EPS *in vitro* to investigate the synergistic effect of water-insoluble EPS and ClyR. The reaction solution was prepared by adding 0.2% (w/v) T-70 glucan and 5% (w/v) sucrose to the 0.2-M sodium phosphate buffer. Gtfs (the method of obtaining was the same as above) and the reaction solution were added to a 24-well plate at a ratio of 1:200 for water-insoluble EPS synthesis at room temperature. After incubating for 24 h, the solution was centrifuged to collect insoluble EPS, which was washed with PBS three times.

### Adsorption assay of water-insoluble EPS with ClyR, PlyCAC, and ClyF

Adsorptive assays were conducted to investigate the correlation between water-insoluble EPS and ClyR. The collected water-insoluble EPS was resuspended in PBS, thoroughly vortexed, and then sonicated in a water bath for 5 min to ensure homogeneous dispersion before gradient dilution and use in subsequent assays. For control experiments, water-insoluble EPS suspensions were subjected to heat treatment (95 °C for 10 min) followed by three PBS washes to remove denatured proteins. Heated water-insoluble EPS samples were then used in adsorption assay in the same manner as untreated water-insoluble EPS. For adsorption assay, 100 µL of diluted water-insoluble EPS solution and 100 µL of ClyR were mixed thoroughly in 1.5-mL centrifuge tubes and incubated at 37 °C for approximately 7 h. After centrifugation (4000 g, 4 °C, 10 min), the supernatant and precipitate were separated by SDS-PAGE (12%) to isolate protein components. The control group consisted of EPS alone to assess background adsorptive levels. BSA was used as a control under identical conditions to determine adsorptive specificity. Similarly, water-insoluble EPS, ClyR, and BSA (the working concentration was 300 μg/mL) were individually added to separate 1.5-mL centrifuge tubes with all other parameters kept constant. The experimental protocols used to investigate the interaction between water-insoluble EPS and PlyCAC or ClyF (the working concentration was 200 μg/mL) were consistently maintained. The adsorptive ratio (%) of proteins by water-insoluble EPS was calculated by quantifying the SDS-PAGE band intensities of the protein (ClyR, PlyCAC, or ClyF) in the supernatant before and after incubation with EPS, using ImageJ software (National Institutes of Health, USA). The ratio was calculated as: [(Intensity of protein in supernatant without EPS − Intensity of protein in supernatant with EPS)/Intensity of protein in supernatant without EPS] × 100%.

### Statistical analysis

Statistical analyses were performed using GraphPad Prism 8.0.2 (GraphPad Software Inc, San Diego, CA, USA). All the experiments were conducted in triplicate and repeated at least three times. Statistical significance between the two groups was determined using Student's *t*-test with a two-tailed *p* < 0.05.

## Results

### Impact of Gtf on ClyR antibacterial activity against *S. mutans*

**Figure 1.**
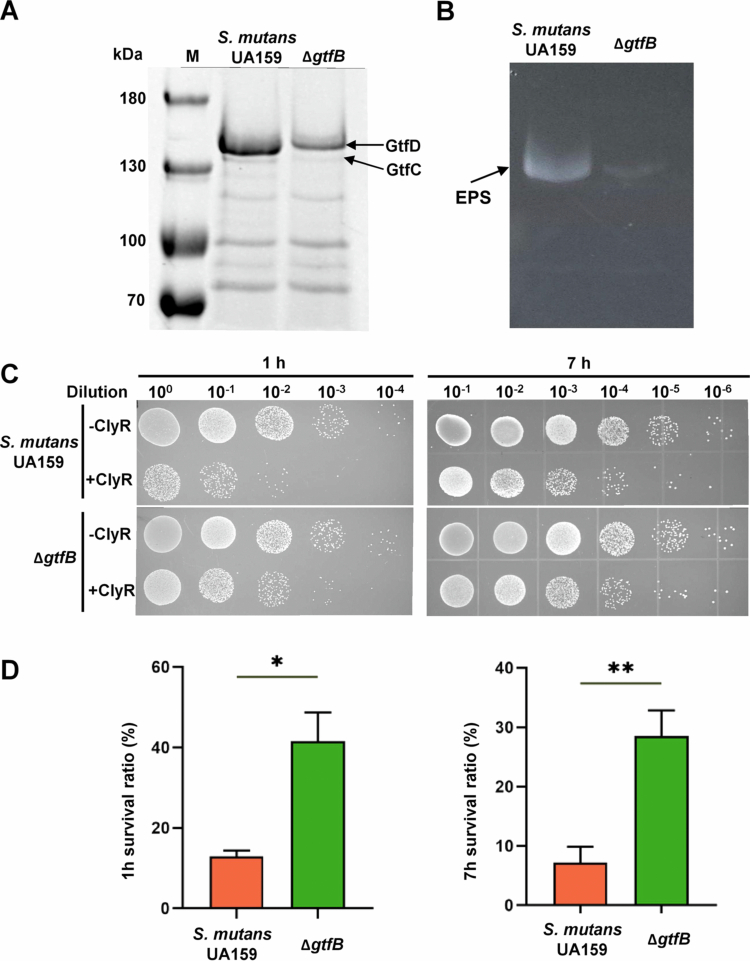
The ∆*gtfB* mutant lacking GtfB exhibited increased resistance to ClyR. (A) Coomassie-stained SDS-PAGE gel showing the Gtfs content in the culture supernatant of *S. mutans* UA159 and the ∆*gtfB* mutant, with arrows indicating GtfC and GtfD bands. (B) Zymogram assay detecting water-insoluble EPS synthesis activity of Gtfs. Bands corresponding to water-insoluble EPS are labeled. (C) 10-fold serial dilutions of bacteria were spotted onto BHIA plates 1 h and 7 h after treatment with and without ClyR, followed by observation and CFU counting after 48 h. (D) Survival rates of *S. mutans* UA159 and ∆*gtfB* were assessed 1 h and 7 h after exposure to ClyR (*p < 0.05, **p < 0.01).

Glucosyltransferases (Gtfs) play a crucial role in forming biofilms and synthesizing water-insoluble EPS in *S. mutans*. To study the effect of different EPS amounts on the lytic activity of ClyR, we utilized the mutant strain lacking GtfB (∆*gtfB*) [19]. This enzyme primarily synthesizes water-insoluble EPS in *S. mutans*. The Coomassie staining and zymogram assay were used to validate the diminished Gtfs content and consequently reduced water-insoluble EPS production in the ∆*gtfB* mutant (Figure 1A and B).

We evaluated the susceptibility of *S. mutans* UA159 and its derivative ∆*gtfB* to ClyR at a concentration of 300 μg/mL. After ClyR exposure, the ∆*gtfB* mutant exhibited a significantly higher survival rate than the UA159 strain (Figure 1C). Specifically, the ∆*gtfB* mutant showed a survival rate of 41.64% after 1 h of ClyR treatment, compared to a 12.99% survival rate for UA159. After 7 h of ClyR treatment, survival rates for ∆*gtfB* and UA159 were 28.55 and 7.23%, respectively (Figure 1D), indicating the enhanced ClyR tolerance in the ∆*gtfB* mutant. These findings reveal an intrinsic link between Gtf-mediated EPS production and the defensive capacity of *S. mutans* against ClyR, indicating that Gtfs might be pivotal determinants of bacteriophage lysin susceptibility.

### Impact of Gtf-mediated EPS production on ClyR antibacterial activity against *S. mutans* biofilm

**Figure 2.**
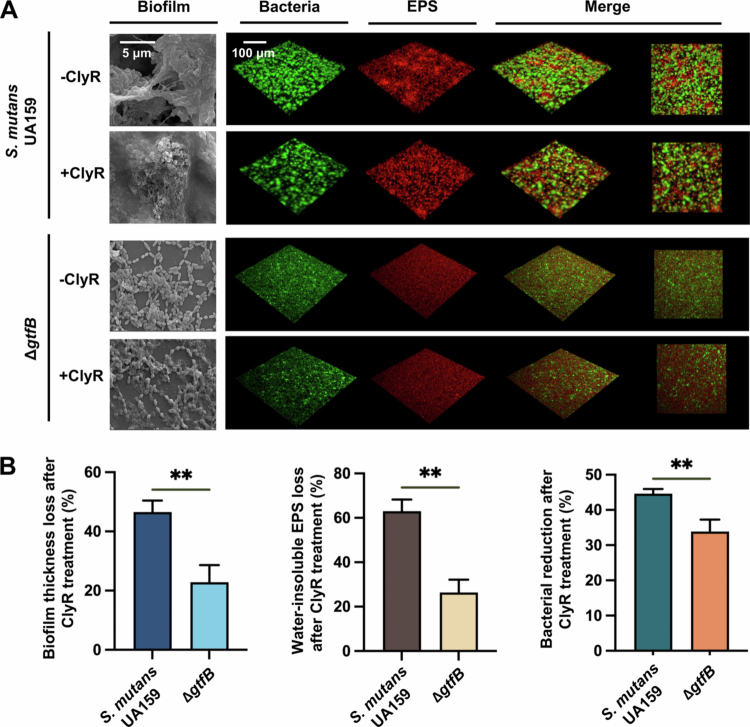
Biomass and structure of *S. mutans* UA159 and *∆gtfB* biofilms after ClyR treatment. (A) Biofilm morphology and biomass of *S. mutans* UA159 and ∆*gtfB* were examined using SEM (× 20,000 magnification) and CLSM. (B) Quantification of biofilm thickness loss, water-insoluble EPS loss, and bacterial reduction after ClyR treatment for both strains (**p < 0.01).

To clarify the role of GtfB-mediated EPS in susceptibility to ClyR, we compared the structural and quantitative changes in *S. mutans* UA159 and Δ*gtfB* biofilms following ClyR treatment using SEM, CLSM, and biomass analysis. As shown in Figure 2A, the Δ*gtfB* mutant formed biofilms with a thinner and less cohesive extracellular matrix than the wild-type strain. Upon ClyR exposure, wild-type biofilms exhibited substantial disruption and loss of bacterial architecture, while the Δ*gtfB* mutant retained more intact biofilm structure. Quantitative analysis revealed that *S. mutans* UA159 biofilms exhibited significantly greater loss of biofilm thickness and water-insoluble EPS after ClyR treatment compared to the Δ*gtfB* mutant (Figure 2B, left and middle panels). Consistently, bacterial reduction after ClyR treatment (Figure 2B, right panel) was also more pronounced in the wild-type strain than in the Δ*gtfB* mutant. These results indicate the essential role of GtfB-dependent EPS production in modulating ClyR susceptibility in *S. mutans*.

### Specific interaction between *S. mutans* water-insoluble EPS and ClyR

**Figure 3.**
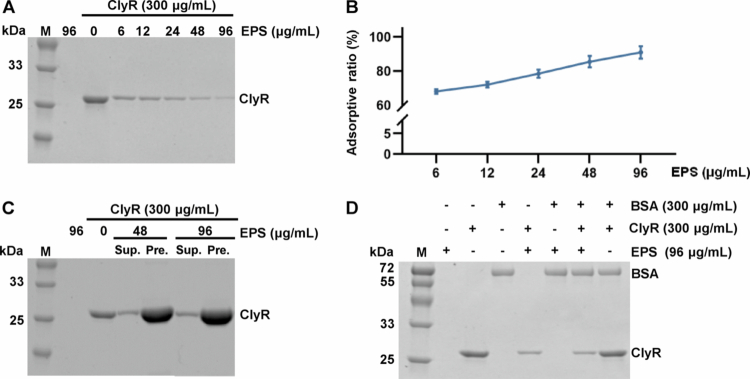
Adsorption experiments of water-insoluble EPS on ClyR. (A) The results of ClyR adsorption by water-insoluble EPS at different concentrations are presented, with lighter bands on the gel indicating lower protein content and darker bands indicating higher protein content in the supernatant. (B) The adsorptive ratio of ClyR by water-insoluble EPS at various concentrations. (C) Protein distribution of supernatant and precipitate after ClyR adsorption by water-insoluble EPS. (Sup. stands for supernatant, and Pre. stands for precipitate.) (D) Determination of the specificity of water-insoluble EPS adsorption of ClyR, with BSA as a control.

We synthesized water-insoluble EPS using *S. mutans* Gtfs extracts *in vitro* to further investigate the potential interactions between water-insoluble EPS and ClyR. A total of 192 μg of dried EPS was resuspended in PBS by vortexing followed by mild sonication to achieve a uniform suspension. Subsequently, dilutions were prepared using a twofold gradient, resulting in final reaction concentrations of 96, 48, 24, 12, and 6 μg/mL, respectively. As shown in Figure 3A–C, increasing concentrations of water-insoluble EPS correlated with a higher percentage of ClyR adsorption from the reaction solution, with adsorption rates of 68.04, 72.05, 78.45, 85.50, and 90.96% for each concentration, respectively, further confirmed by analyzing the precipitates formed after centrifugation, which showed ClyR accumulation. BSA was used as a control protein to investigate the specificity of water-insoluble EPS binding towards ClyR. The results indicated that BSA was not adsorbed by EPS. Similarly, when both BSA and ClyR were mixed with water-insoluble EPS simultaneously, only the amount of ClyR decreased while BSA remained unaffected in the supernatant (Figure 3D). To further exclude the possibility of residual protein contaminants, we performed adsorption assays using heat-treated water-insoluble EPS. As shown in Figure S1, increasing concentrations of heated water-insoluble EPS also resulted in a dose-dependent adsorption of ClyR, consistent with untreated water-insoluble EPS. These experimental results suggest that the water-insoluble EPS produced by *S. mutans* Gtfs extracts could specifically interact with ClyR.

### Specific binding of *S. mutans* water-insoluble EPS to the catalytic domain of ClyR

**Figure 4.**
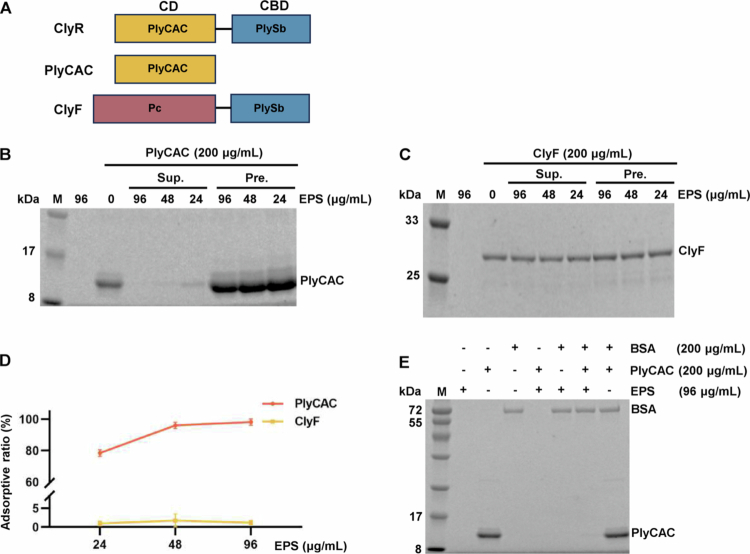
Water-insoluble EPS adsorption experiments of ClyR derivatives. (A) The structures of ClyR and ClyF are depicted. ClyR is composed of PlyCAC and PlySb, whereas ClyF consists of Pc and PlySb. (B) The impact of different concentrations of water-insoluble EPS on the adsorption of PlyCAC was investigated, and the distribution of proteins in the supernatant and precipitate post-adsorption was analyzed. (Sup. stands for supernatant, and Pre. stands for precipitate.) (C) The distribution of ClyF in the supernatant and precipitate after water-insoluble EPS-mediated adsorption at various concentrations is presented to illustrate the results of ClyF adsorption by water-insoluble EPS. (D) The adsorptive ratio of PlyCAC and ClyF by water-insoluble EPS at various concentrations. (E) The adsorption specificity of water-insoluble EPS towards PlyCAC was revealed through controlled experiments, with BSA serving as a control.

Previous studies have established that PlyCAC functions as the CD of ClyR, while ClyF shares the same CBD but possesses a different CD compared to ClyR (Figure 4A) [12,17]. We conducted the following adsorption experiments to further explore the specific domain of ClyR that water-insoluble EPS interacts with. The results demonstrated a significant reduction of PlyCAC in the supernatant and its accumulation in the precipitates as water-insoluble EPS concentration increased (Figure 4B). Conversely, ClyF was not effectively adsorbed by water-insoluble EPS (Figure 4C). Quantitative analysis showed the high adsorption efficiency of PlyCAC, with average adsorption ratios of 98.80, 95.77, and 78.50%, respectively (Figure 4D), indicating a strong adsorptive capability of water-insoluble EPS to PlyCAC. Additionally, BSA was used as a control, and the results demonstrated that upon simultaneous addition of PlyCAC and BSA to water-insoluble EPS, only PlyCAC was adsorbed by water-insoluble EPS, indicating a specific interaction between water-insoluble EPS and PlyCAC (Figure 4E). These findings suggest that the water-insoluble EPS produced by *S. mutans* Gtfs extracts could specifically interact with the catalytic domain of ClyR.

## Discussion

This study investigated the antibacterial activities of ClyR against *S. mutans* and its Δ*gtfB* derivative and explored the underlying factors contributing to the variability in the susceptibility of *S. mutans*. Interestingly, water-insoluble EPS synthesized by Gtfs mediated the antibacterial activity of ClyR against *S. mutans*. These findings provide novel insights into the interactions between ClyR and water-insoluble EPS, advancing our understanding of potential therapeutic strategies against infectious diseases.

The lyase secreted by bacteriophages enzymatically degrades the bacterial cell wall, facilitating the release of fully assembled phage particles. Delisle ingeniously incorporated the lyase derived from M102, e10 and f1, three virulent *S. mutans* bacteriophages that were isolated and identified in 1993 and belong to the Longtail family, into toothpaste and mouthrinse formulations as a preventive measure against dental caries and other oral diseases [20,21]. ClyR, identified by Yang through bioengineering techniques *in vitro*, is the first phage lyase reported to exhibit antagonistic activity against all known *S. mutans* serotypes [12]. However, the lytic activity of ClyR against various clinical strains of *S. mutans* is significantly inconsistent [16], posing a challenge to the clinical application of ClyR and necessitating a deeper understanding of the factors influencing bacterial resistance or sensitivity to these antimicrobial agents.

Bacterial biofilm is a unique mode of existence of microorganisms in nature. Compared with planktonic cells, bacterial biofilms are more adaptable to complex living environments [22]. In the classical four-factor theory of dental caries, microorganisms are the primary factor, and dental plaque biofilm is the carrier and form of microbial cariogenicity. *S. mutans* is the main cariogenic bacteria in biofilm formation [2,23]. Glucosyltransferase use sucrose to synthesize glucans [24]. The production of EPS, particularly water-insoluble EPS, is a crucial cariogenic virulence factor of *S. mutans* [25], which facilitates bacterial adhesion, binding site formation, and biofilm matrix development [26]. Similar trends were also observed in the ∆*gtfB* mutant, which was engineered in our previous study [19], characterized by a single-site mutation with less Gtfs and water-insoluble EPS content. These findings suggest that the Gtfs-mediated water-insoluble EPS content within biofilms might be crucial in mediating the strain's resistance to ClyR cleavage.

The water-insoluble EPS, a polysaccharide synthesized by Gtfs from *S. mutans*, was synthesized *in vitro* using native Gtfs extracts and sucrose for functional assessment of its interaction with ClyR in this study. The water-insoluble EPS was then collected, co-incubated with ClyR, and proteins remaining in the supernatant were analyzed using 12% SDS–PAGE electrophoresis. There was a gradual decrease in ClyR concentration in the supernatant with an increasing concentration of water-insoluble EPS, accompanied by a significant accumulation of ClyR in the precipitates. The results demonstrated the ability of water-insoluble EPS to adsorb ClyR, leading to its accumulation in deposits. Many studies have shown that phage tail silk proteins can specifically bind to and degrade capsular polysaccharides, EPS, and lipopolysaccharides on bacterial surfaces [27], possibly explaining the precise structural specificity exhibited by ClyR in its recognition of water-insoluble EPS derived from *S. mutans*. This suggests that the production of water-insoluble EPS by *S. mutans* may be crucial in mediating its response to ClyR treatment. In the biofilm phenotype assays, both *S. mutans* UA159 and the Δ*gtfB* mutant were grown in BHI supplemented with 1% sucrose to promote EPS production. Our results show that the Δ*gtfB* mutant forms biofilms with a significantly reduced EPS matrix and displays greater resistance to ClyR-mediated lysis compared to the WT strain. After ClyR treatment, the reduction in bacterial biomass and the disruption of biofilm structure were much less pronounced in the Δ*gtfB* mutant than in the WT, supporting the conclusion that GtfB-mediated EPS production plays a key role in modulating ClyR susceptibility. We also acknowledge that planktonic bactericidal assays performed without sucrose supplementation may partially reflect glucan-independent effects of *gtfB* deletion, which warrants further investigation.

Furthermore, the specific interaction between *S. mutans* water-insoluble EPS and the catalytic domain of ClyR provides a novel insight into the molecular mechanisms underlying the bactericidal action of phage lytic enzymes. The preferential interaction of water-insoluble EPS with the catalytic domain of ClyR highlights the critical role of this domain in mediating interactions with the biofilm matrix and facilitating the lytic activity of ClyR. PlyCAC, the catalytic domain of ClyR, corresponds to the CHAP domain of the PlyC lysin (amino acids 314−465). The CHAP domain, which is the enzymatic catalytic domain of PlyC lysin [28], is common to many amidases, including some peptidoglycan hydrolases [29], and mediates nucleophilic attack by using catalytic cysteine residues [30]. Crucial CHAP binding sites for water-insoluble EPS might exist to facilitate the subsequent adsorption of ClyR, warranting further investigation.

The insights gained from this study have profound implications for developing novel therapeutic strategies for dental caries. Given that insoluble EPS produced by *S. mutans* can coat not only itself but also neighboring species within dental biofilms, it is plausible that the presence of glucans could increase the local concentration and retention of ClyR in the biofilm matrix, potentially exposing other bacteria embedded within the EPS to its lytic activity. Whether this would lead to effective lysis of other species may depend on the compatibility of their cell wall structures with the catalytic specificity of ClyR. In addition, targeting the biofilm's water-insoluble EPS and enhancing the lytic activity of bacteriophage enzymes might help overcome the limitations posed by the variable susceptibility of *S. mutans*. Future research should explore the molecular basis of water-insoluble EPS-lysozyme interactions to develop engineered lytic enzymes with improved efficacy against cariogenic biofilms. Additionally, the potential of combining bacteriophage lysozymes with other antimicrobial agents or treatment modalities warrants further exploration to maximize therapeutic outcomes.

Our study highlights the therapeutic potential of bacteriophage chimeric lysozyme ClyR, in combating dental caries by interacting with water-insoluble EPS of *S. mutans*. The water-insoluble EPS in *S. mutans* biofilms not only acts as a structural barrier but may also play a facilitating role in localizing ClyR and enhancing its lytic activity. Our data suggest that the presence of abundant EPS, which specifically binds to the catalytic domain of ClyR, may increase the local retention of the enzyme within the biofilm, thereby promoting more efficient lysis of wild-type *S. mutans* compared to the Δ*gtfB* mutant. This may explain why the Δ*gtfB* mutant, despite having less EPS for ClyR binding, is more resistant to lysis, as the sparse matrix is less effective at concentrating the enzyme near the bacterial cells. Collectively, this research contributes valuable knowledge towards developing novel antimicrobial strategies against dental caries and other biofilm-associated infections by providing new insights into the mechanisms underlying bacterial resistance and sensitivity to phage-derived lytic enzymes.

## Supplementary Material

Supplementary material Figure S1. Adsorption experiments of heated water-insoluble EPS on ClyR. Heated water-insoluble EPS (95 °C, 10 min, followed by PBS washes) was incubated with ClyR at increasing concentrations. The results demonstrate that increasing concentrations of heated water-insoluble EPS induce a dose-dependent adsorption of ClyR, consistent with untreated water-insoluble EPS.

Supplementary material Table S1 Primers used in this study.

Supplementary material Figure_S1
