# Supplementary material for: Water-insoluble exopolysaccharide synthesized by glucosyltransferases mediates the antibacterial activity of ClyR against Streptococcus mutans
Source: J Oral Microbiol. 2025 Oct 9;17(1):2566894. doi: 10.1080/20002297.2025.2566894 (PMC12517413; doi:10.1080/20002297.2025.2566894)
Supplement: Supplementary material — Figure S1. Adsorption experiments of heated water-insoluble EPS on ClyR. Heated water-insoluble EPS (95 °C, 10 min, followed by PBS washes) was incubated with ClyR at increasing concentrations. The results demonstrate that increasing concentrations of heated water-insoluble EPS induce a dose-dependent adsorption of ClyR, consistent with untreated water-insoluble EPS. [file ZJOM_A_2566894_SM3841.docx]

**Supplementary information**

**
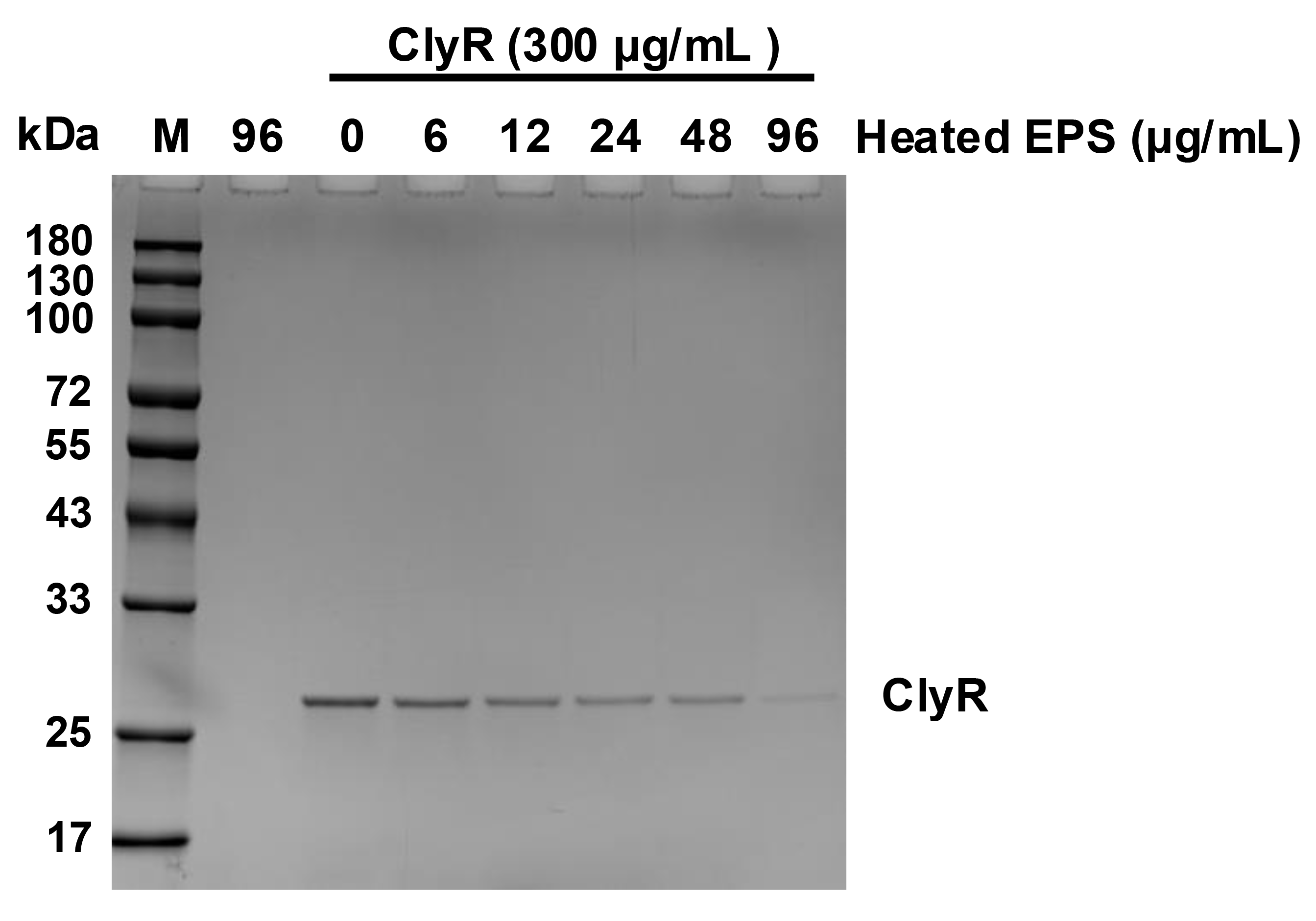
**

**Figure S1. Adsorption experiments of heated water-insoluble EPS on ClyR.** Heated water-insoluble EPS (95 °C, 10 min, followed by PBS washes) was incubated with ClyR at increasing concentrations. The results demonstrate that increasing concentrations of heated water-insoluble EPS induce a dose-dependent adsorption of ClyR, consistent with untreated water-insoluble EPS.
