# Supplementary figures and images for: Water-insoluble exopolysaccharide synthesized by glucosyltransferases mediates the antibacterial activity of ClyR against Streptococcus mutans
Source: J Oral Microbiol. 2025 Oct 9;17(1):2566894. doi: 10.1080/20002297.2025.2566894 (PMC12517413; doi:10.1080/20002297.2025.2566894)

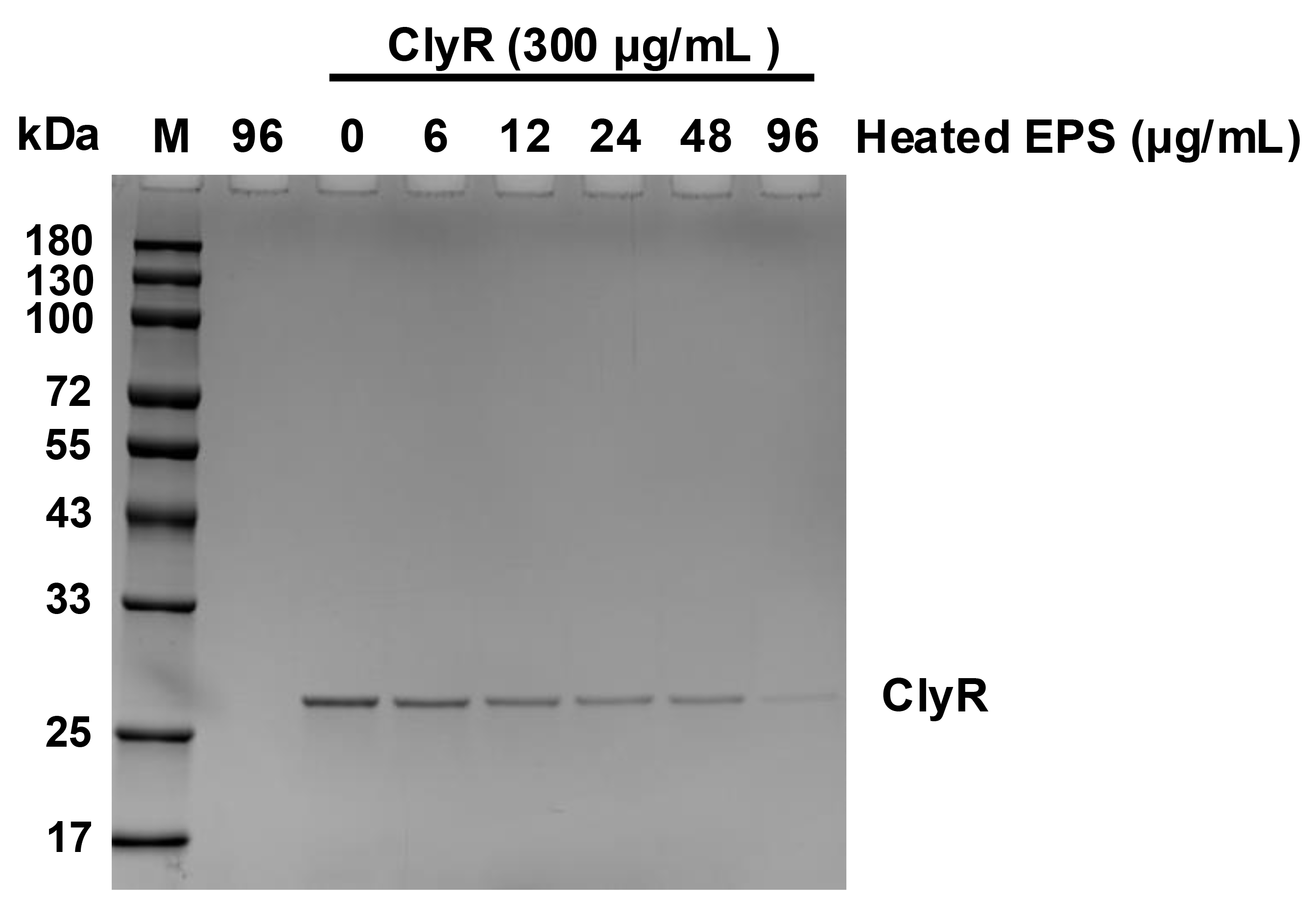

Supplement: Supplementary material — Figure_S1 [file ZJOM_A_2566894_SM0529.tif]
